# Effects of digital media technology-driven film and television scene design on audience immersion and narrative cognition: an eye-tracking experiment

**DOI:** 10.3389/fpsyg.2026.1919138

**Published:** 2026-10-21

**Authors:** Lingling Shen

**Affiliations:** School of Art and Design, Anhui University of Applied Sciences, Wuhu, China

**Keywords:** audience immersion, digital media technology, eye-tracking, film and television scene design, narrative cognition, visual attention

## Abstract

Digital media technology increasingly shapes film and television scene design, but evidence remains limited on how technology-driven scene treatments influence viewers' attention, immersion, and narrative cognition within the same experimental framework. This study used a randomized between-groups design with 360 valid adult participants (180 per condition). Participants viewed matched low- and high-digital-media versions of the same narrative scene and completed an immersion measure and an objective narrative-cognition test while eye-tracking data were recorded. Visual attention to pre-defined key narrative areas of interest (AOIs) was examined as a potential mediating mechanism. Controlling for gender, age, viewing frequency, digital-media familiarity, and prior VR experience, the high-digital-media version was associated with greater immersion (*B* = 1.085, SE = 0.048), greater narrative cognition (*B* = 1.208, SE = 0.056), and greater attention to key narrative AOIs (*B* = 0.109, SE = 0.007); the three models accounted for 59.1, 57.7, and 44.0% of the variance respectively. Bootstrapped indirect effects were consistent with partial mediation by attention to narrative AOIs for immersion (a × *b* = 0.170, 95% CI [0.094, 0.248]) and narrative cognition (a × *b* = 0.225, 95% CI [0.126, 0.324]). Replacing fixation proportion with total fixation duration reproduced the pattern for immersion (a × *b* = 0.136, 95% CI [0.004, 0.275]) but not for narrative cognition (a × *b* = 0.020, 95% CI [−0.132, 0.167]). These findings suggest that digital media technology can support narrative experience when technological features are coordinated with story-relevant visual cues rather than functioning as isolated spectacle. Because both conditions were implemented in one matched pair of clips, condition is fully confounded with stimulus identity (stimulus sampling), and the results describe these two clips rather than digital media scene design as a class. Because the sample also consisted mainly of young adults, the findings should be generalized cautiously. Replication with multiple independently validated stimulus sets analyzed with stimulus as a random factor, additional genres, and broader participant groups is warranted.

## 1 Introduction

Digital media technology is continuously reshaping the expression of film and television scene design (Swords and Willment, 2024). The traditional film and television scenes mainly rely on real scene construction, lens scheduling, lighting layout and post synthesis to complete the spatial presentation, while the intervention of virtual image, real-time rendering, dynamic light and shadow, spatial sound and immersive vision system gradually makes the film and television scenes have stronger spatial extensibility, sensory wrapping and narrative organization ability (Bodini et al., 2024; Zhang and Weber, 2023). In this change, the scene is no longer just the background of the characters' actions, but an important medium for the audience to understand the plot, perceive the atmosphere and form the viewing experience. The audience's attention distribution to characters, props, space entrances, light and shadow changes and sound sources may be directly related to their immersion experience and narrative understanding (Peacock et al., 2023a; Cannavò et al., 2024).

Research on technology-enhanced screen production has established the growing role of virtual production, immersive cinematography, and digitally constructed environments (Dooley, 2020; Carpio and Birt, 2022; Nielsen and Sheets, 2021). However, the present question is not simply whether digital scenes appear more technologically sophisticated. Film- and media-psychology research shows that narrative engagement depends on attentional focus, narrative understanding, emotional engagement, and narrative presence (Green and Brock, 2000; Busselle and Bilandzic, 2009), while film-specific eye-tracking studies demonstrate that editing, motion, and task demands can strongly synchronize or redirect viewers' gaze (Smith, 2012; Smith and Mital, 2013; Loschky et al., 2015; Hutson et al., 2022; Ding and Chen, 2022). This literature makes the central problem more precise: a visually salient digital treatment may improve immersion only when it guides attention toward information that supports the unfolding narrative. Accordingly, the present study examines scene design, key-narrative-area attention, immersion, and narrative cognition within one experimental framework rather than inferring audience effects from design features alone.

This study therefore tests a controlled comparison between low- and high-digital-media scene conditions. Eye-tracking is used to quantify visual attention to key narrative areas, while immersion and narrative cognition are measured with self-report and objective test items, respectively. The design is intended to distinguish three questions: whether the scene manipulation changes the audience experience, whether it changes allocation of visual attention, and whether visual attention statistically accounts for part of the association between scene condition and audience outcomes.

The contribution is three fold. First, the study treats digital media technology-driven scene design as an empirically testable audience-experience problem rather than only an artistic or technical proposition. Second, it combines eye-tracking with subjective and objective outcome measures, reducing reliance on questionnaire-only evidence. Third, it connects production choices to film-cognition research on attentional guidance and narrative engagement, thereby providing a more operational basis for evaluating whether virtual-space, lighting, visual-effect, and sound treatments support rather than compete with narrative cues. The scope of the demonstration is correspondingly bounded: because the manipulation is realized in one matched pair of clips, the study provides a controlled within-pair test rather than a general estimate of the effect of digital media scene design.

## 2 Literature review and research hypotheses

### 2.1 Digital media technology-driven film and television scene design

Digital media technology-driven film and television scene design refers to the integrated use of virtual imagery, real-time rendering, dynamic lighting, spatial sound, digital visual effects, and related production techniques to organize screen space and audience perception (Swords and Willment, 2024; Bodini et al., 2024; Zhang and Weber, 2023; Dooley, 2020; Cannavò et al., 2024). In narrative media, these techniques are most consequential when they structure where viewers look and how they connect characters, props, spatial transitions, and causal events. Thus, the relevant theoretical issue is not technological intensity by itself, but the coordination of technological treatment with narrative information.

### 2.2 Audience immersion and narrative engagement

Audience immersion overlaps with the established concepts of transportation and narrative engagement. Transportation emphasizes absorption, imagery, affect, and attentional focus (Green and Brock, 2000), whereas narrative engagement distinguishes narrative understanding, attentional focus, emotional engagement, and narrative presence and has been validated with film and television viewers (Busselle and Bilandzic, 2009). Recent work using film and television clips further shows that self-reported immersion can covary with continuous behavioral and physiological indicators of attention (Hammond et al., 2023). In the present study, immersion is therefore treated as a multidimensional viewing state rather than as a direct proxy for visual realism.

### 2.3 Audience narrative cognition

Narrative cognition concerns how viewers construct and update event models linking plot events, character relations, causal structure, spatial changes, and scene-relevant cues (Zacks, 2020; Cohn and Magliano, 2020; Loschky et al., 2020; Cohn, 2020; Franklin et al., 2020; Laubrock and Dunst, 2020). Film-specific research is particularly important here. Eye-tracking studies have shown that viewers can display highly similar gaze patterns even when their narrative comprehension differs, while narrative context can still guide attention under conditions that permit stronger top-down control (Loschky et al., 2015; Hutson et al., 2022). These findings caution against treating gaze as equivalent to comprehension and support the present use of a separate objective narrative-cognition measure.

### 2.4 Eye-tracking and visual attention during film viewing

Eye-tracking provides time-resolved measures of fixation location and duration and is therefore well suited to examining whether viewers attend to story-relevant screen regions. General visual-search principles remain useful (Niehorster et al., 2020a,b; Hessels, 2020; Peacock et al., 2023b; Franconeri et al., 2021; Wolfe, 2021; Harris and Remington, 2020; Carter and Luke, 2020; Wolfe, 2020), but film viewing has distinctive dynamics. Cinematic continuity and editing can guide attention across cuts (Smith, 2012), dynamic scenes can produce substantial inter-viewer attentional synchrony (Smith and Mital, 2013), and film-specific experiments show that comprehension, task, motion, and affect can alter or constrain gaze allocation (Loschky et al., 2015; Hutson et al., 2022; Ding and Chen, 2022). Because eye-tracking results are sensitive to hardware, calibration, pre-processing, fixation classification, data loss, and AOI construction, the reporting framework follows the dimensions emphasized in eye-tracking best-practice guidance (Carter and Luke, 2020).

### 2.5 Research hypotheses

The literature suggests that digital scene treatments may influence audience outcomes through both direct perceptual experience and the allocation of attention to story-relevant information. The hypotheses are therefore stated separately for immersion, narrative cognition, visual attention, and mediation. Because the manipulation is implemented in a single matched pair of clips, each hypothesis is stated and tested with respect to this stimulus pair; none of them is a statement about digital media scene design as a class.

H1: Digital media technology-driven film and television scene design is positively associated with audience immersion. The hypothesis is grounded in transportation and narrative-engagement research, which identifies attentional focus, narrative presence, and emotional engagement as central components of immersive experience (Green and Brock, 2000; Busselle and Bilandzic, 2009; Hammond et al., 2023).
H2: Digital media technology-driven film and television scene design is positively associated with audience narrative cognition. This expectation is consistent with film-cognition research showing that narrative context and event models shape how viewers interpret screen information, even when gaze is strongly constrained by cinematic presentation (Loschky et al., 2015; Hutson et al., 2022).
H3: Digital media technology-driven film and television scene design is positively associated with visual attention to key narrative AOIs. Cinematic editing, motion, lighting, and task-relevant cues can guide and synchronize gaze during dynamic viewing (Smith, 2012; Smith and Mital, 2013; Ding and Chen, 2022). The prediction is therefore that a scene treatment that coordinates digital elements with story-relevant targets will increase attention to those targets rather than merely increasing diffuse visual salience.
H4: Visual attention to key narrative AOIs mediates the association between digital media technology-driven scene design and both audience immersion and narrative cognition. This hypothesis does not assume that gaze and comprehension are interchangeable; rather, it tests whether attention to explicitly defined narrative targets accounts for part of the relationship between the experimental condition and the two outcome measures (Busselle and Bilandzic, 2009; Loschky et al., 2015; Hutson et al., 2022; Ding and Chen, 2022; Hammond et al., 2023).

## 3 Research methods

### 3.1 Participants, stimuli, and experimental design

The study used a randomized between-groups experiment with adults aged 18–35 who had basic film- and television-viewing experience. Participants were recruited through university public courses, film- and media-related communities, and online announcements. Eligibility required normal or corrected-to-normal vision and the ability to complete eye-tracker calibration; individuals reporting severe color-vision problems or substantial visually induced dizziness were excluded. Participants were randomly assigned to the low-digital-media or high-digital-media scene condition before viewing.

The stimuli were two matched versions of the same underlying narrative source (Clip_L_03 and Clip_H_03). Their durations were 240.5 and 241.2 s, respectively, and both were presented at 1,920 × 1,080 resolution and 24 fps with the same 32-shot edit order, Dialogue Script Ver. 3.1, character actions, and key narrative events involving the locket, letter, and door. The low-digital-media version used minimal set extension, static three-point lighting, practical effects, and a stereo mixdown; the high-digital-media version used a dynamic 3D environment, interactive CGI lighting, particle and atmospheric visual effects, and 5.1 surround with object audio. Integrated dialogue loudness was closely matched (−24.0 vs. −24.1 LUFS). In a 40-participant pilot, narrative clarity/comprehensibility did not differ between versions (5.43 ± 1.12 vs.5.39 ± 1.08), *t* (38) = 0.11, *p* = 0.91, *d* = 0.04; the familiarity/interest/valence composite also did not differ (5.51 ± 1.05 vs.5.45 ± 0.98; all *p* > 0.60). Supplementary Appendix A provides the complete construction and equivalence record. Because both conditions were realized in a single matched pair of clips that share the same narrative source, shot order, dialogue, props, and key events, the experimental condition is fully confounded with stimulus identity (stimulus sampling). The stimulus pair is therefore treated as a fixed factor: the estimates reported below describe the difference between Clip_L_03 and Clip_H_03, they cannot be generalized to digital media scene design as a class or to other narratives, genres, and production budgets, and they are conditional on this pair in the sense that treating stimulus as a random factor would require multiple independently constructed stimulus sets (Judd et al., 2012).

A total of 376 participants were initially recruited. Screening excluded records with failed or high-error eye-tracker calibration, insufficient valid gaze data, interrupted or clearly distracted viewing, abnormal viewing duration, or invalid questionnaire/narrative-test responses. Sixteen records were excluded, leaving 360 valid participants: 180 in the low-digital-media condition and 180 in the high-digital-media condition. Group distributions for gender, age, viewing frequency, digital-media familiarity, and prior VR experience are summarized in Table 1.

**Table 1:** Sample data sources and experimental grouping.

Project	Low-digital-media scene group	High-digital-media scene group	Total
Experimental condition	Conventional scene treatment with limited digital lighting, virtual-space, visual-effect, and spatial-sound enhancement	Enhanced virtual-space, dynamic-lighting, digital-visual-effect, and spatial-sound treatment	Experimental design between two groups
Initial number of samples	188	188	376
Number of rejected samples	8	8	16
Number of effective samples	180	180	360
Sex ratio	83 males and 97 females	91 males and 89 females	174 males and 186 females
Average age	25.31	24.97	25.14
Film and television viewing frequency	4.91	4.87	4.89
Familiarity with digital media	4.87	4.80	4.84
VR experience	0.46	0.45	0.45

The study was conducted in accordance with the Declaration of Helsinki and received approval from the Institutional Review Board of the School of Art and Design, Anhui Business College (IRB-HMS-2025-0621). All participants were adults aged 18–35 and provided their own informed consent before participation; no legal-guardian or next-of-kin consent was sought or required. Participants were informed about the study purpose, procedures, data use, confidentiality protections, and their right to withdraw without penalty. Questionnaire, narrative-cognition, and eye-tracking records were anonymized for analysis.

#### 3.1.1 Eye-tracking reporting

Gaze was recorded binocularly at 1,200 Hz with a Tobii Pro Spectrum, which is a screen-based remote (non-wearable) eye tracker, while stimuli were displayed on a 25-inch, 1,920 × 1,080 LCD monitor at a viewing distance of 65 cm with a chin rest. No head-mounted display and no headset-mounted eye tracker were used at any stage of the study; the apparatus is a tabletop, screen-based tracker, so the term “headset” does not describe it. A 9-point calibration was accepted when average validation error was below 0.5; drift was checked before each clip and recalibration was performed when drift exceeded 0.8. Fixations were classified with the I-VT algorithm using a 30/s velocity threshold and a 60-ms minimum duration. Missing gaps of no more than 75 ms were linearly interpolated, and participants were required to have more than 70% valid gaze samples. Mean data loss was 5.82% in the low-digital-media condition and 6.15% in the high-digital-media condition; the difference was not significant, *t* (358) = −0.85, *p* = 0.396, *d* = −0.09. Supplementary Appendix B reports the complete acquisition, calibration, pre-processing, and data-quality checklist in accordance with best-practice guidance (Carter and Luke, 2020).

### 3.2 Variable measurement

The independent variable, digital media scene design (DMSD), represents experimental condition: 0 = low-digital-media scene and 1 = high-digital-media scene. The variables and their measurements are summarized in Table 2. The manipulation-check measure (DMSD_*P*) captures perceived digital-media technology, virtual-space realism, digital lighting, and scene-technology integration. Audience immersion and narrative cognition (NC) are the two outcome variables, and gender, age, film/television viewing frequency (MovieFreq), digital-media familiarity (DMF), and prior VR experience (VRExp) are included as control variables.

**Table 2:** Summary of variable measurement.

Variable type	Variable name	Variable abbreviation	Measurement method and assignment description
Independent variable	Film and television scene design driven by digital media technology	DMSD	Experimental condition: 0 = low-digital-media scene; 1 = high-digital-media scene.
Manipulation-check variable	Digital media scene design perception	DMSD_*P*	Continuous perceived-treatment score on a 7-point metric, recorded as a participant-level value; the higher the score, the stronger the perceived digital-media scene treatment
Mediating variable	Visual attention to key narrative AOIs	VA	Proportion of viewing time spent inside the pre-defined key narrative AOIs, expressed on a 0–1 scale, computed from the frame-level fixation record
Robustness variable	Total fixation duration in key narrative AOIs	VA_Dur	Eye-tracking measure used as the robustness alternative to fixation proportion
Dependent variable	Audience immersion	Immersion	Mean of the immersion items on a 7-point scale, recorded as a continuous participant-level score; the higher the score, the stronger the sense of immersion
Dependent variable	Audience narrative cognition	NC	Continuous score on the narrative-cognition test (plot recall, character relationships, causal understanding, scene-clue recognition) on a 7-point metric; the higher the score, the higher the level of narrative cognition
Control variable	Gender	Gender	Male is assigned a value of 1 and female is assigned a value of 0
Control variable	Age	Age	Continuous variable, measured by age
Control variable	Film and television viewing frequency	MovieFreq	Continuous score on a 7-point viewing-frequency scale, recorded as a participant-level value; the higher the score, the more frequent the viewing
Control variable	Familiarity with digital media	DMF	Continuous score on a 7-point familiarity scale, recorded as a participant-level value; the higher the score, the greater the familiarity
Control variable	VR experience	VRExp	Binary indicator of prior virtual-reality experience: 0 = none, 1 = one or more prior VR experiences

Visual attention (VA) is derived from fixation data within pre-defined key narrative areas of interest (AOIs): character faces, key props, spatial entrances, narrative lighting regions, and localized sound-source regions. AOIs were defined on the shot-by-shot reference structure that the two versions have in common (identical 32-shot edit order, identical framing, identical character actions, and identical key narrative events) and were therefore anchored to the same screen regions in both conditions, so that each category denotes the same narrative element in both versions. The manipulation changes what occurs inside those regions rather than which regions are designated: a static three-point lighting set-up becomes a moving, interactive light beam within the same narrative-lighting region, and a stereo mixdown becomes object-based spatial audio for the same visible sound source. The operational definitions and the frame-by-frame coding rules for each category are listed in Supplementary Appendix C1 and were applied to the shared reference structure. The primary measure remains fixation proportion within key narrative AOIs; total fixation duration (VA_Dur) is retained as a robustness measure, and its descriptive statistics are reported in Table 3. Supplementary Appendix C reports the operational definitions, the within-AOI fixation shares by condition, and the AOI materials that accompany the submission.

**Table 3:** Descriptive statistical results of main variables.

Variable	Minimum value	Maximum value	Mean (*M*)	Standard deviation (SD)
DMSD	0.000	1.000	0.5000	0.5007
DMSD_P	2.103	7.000	4.5630	1.5769
VA	0.311	0.748	0.5234	0.0831
VA_Dur	64.977	130.854	96.5139	14.6628
Immersion	3.112	6.758	5.0180	0.7084
NC	2.807	6.962	4.8684	0.7998
Gender	0.000	1.000	0.4833	0.5004
Age	18.000	35.000	25.1361	3.7769
MovieFreq	1.000	7.000	4.8910	1.2206
DMF	1.260	7.000	4.8353	1.1875
VRExp	0.000	1.000	0.4528	0.4985

Immersion is measured on a 7-point scale covering presence, attentional engagement, emotional involvement, and viewing coherence. Narrative cognition is measured with objective items on plot recall, character relationships, causal understanding, and scene-clue recognition. The analysis therefore keeps subjective immersion, objective narrative cognition, and eye-tracked visual attention as distinct constructs rather than treating any one measure as a substitute for the others. Because narrative cognition is assessed with an objective test rather than with a second self-report scale, the association between condition and narrative cognition cannot be attributed to shared method variance between predictor and outcome (Podsakoff et al., 2003).

### 3.3 Empirical model

To align the empirical tests with H1–H4, three regression equations are specified. The first estimates the association between DMSD and each audience outcome, the second estimates the association between DMSD and visual attention, and the third adds visual attention to the outcome model to evaluate the indirect pathway. Control variables are entered consistently where indicated. Indirect effects are evaluated with 5,000 bootstrap resamples with seed 12345, and the standard error of the product of coefficients is also reported for comparison (Sobel, 1982; Preacher and Hayes, 2008). Complete model-fit, collinearity, mediation-path, and bootstrap diagnostics are reported in Supplementary Appendix D.


$$\begin{array}{l} \text{Immersio}{\text{n}}_{i}={\text{α}}_{0}+{\text{α}}_{1}\text{DMS}{\text{D}}_{i}+{\text{α}}_{2}\text{Control}{\text{s}}_{i}+\;{\text{ε}}_{i}\\ \text{ N}{\text{C}}_{i}={\text{β}}_{0}+{\text{β}}_{1}\text{DMS}{\text{D}}_{i}+{\text{β}}_{2}\text{Control}{\text{s}}_{i}+{\text{ε}}_{i} \end{array}$$ (1)

Equation (2) tests whether the experimental scene condition predicts visual attention to key narrative AOIs.


$$\begin{array}{l} \text{V}{\text{A}}_{i}={\text{γ}}_{0}+{\text{γ}}_{1}\text{DMS}{\text{D}}_{i}+{\text{γ}}_{2}\text{Control}{\text{s}}_{i}+{\text{ε}}_{i} \end{array}$$ (2)

Equation (3) estimates immersion or narrative cognition (Y_*i*_) while including visual attention as the mediator. The indirect effect is the product of the DMSD → VA and VA → Y paths and is evaluated with a bootstrap confidence interval and with the asymptotic standard error of that product.


$$\begin{array}{l} {\text{Y}}_{i}={\text{δ}}_{0}+{\text{δ}}_{1}\text{DMS}{\text{D}}_{i}+{\text{δ}}_{2}\text{V}{\text{A}}_{i}+{\text{δ}}_{3}\text{Control}{\text{s}}_{i}+{\text{ε}}_{i} \end{array}$$ (3)

In (1)–(3), Immersion_*i*_ is the immersion score for participant i; NC_*i*_ is narrative cognition; DMSD_*i*_ is the randomized scene-condition indicator; VA_*i*_ is fixation proportion within key narrative AOIs; Controls_*i*_ denotes gender, age, MovieFreq, DMF, and VRExp; and ε_*i*_ is the residual error term. For the mediation models, Y_*i*_ denotes either immersion or narrative cognition. This notation is used consistently in all subsequent tables.

The analysis combines coefficient estimation with product-of-coefficients testing of the indirect effect. To maintain internal statistical consistency, the main regression tables report coefficient estimates, standard errors, sample sizes, *R*^2^, adjusted *R*^2^, omnibus *F* tests, and maximum variance inflation factors. Supplementary Appendix D consolidates the corresponding model-fit and mediation verification results from the participant-level re-estimation.

## 4 Empirical analysis

### 4.1 Manipulation test, descriptive statistics and correlation analysis

This section first evaluates whether participants perceived the intended difference between the two scene conditions, then summarizes the main variables and their zero-order correlations. The manipulation check is treated separately from the substantive outcome analyses because an intentionally strong manipulation can yield a very large standardized difference without implying a similarly large effect on immersion or narrative cognition.

Table 4 reports the manipulation check. Participants in the high-digital-media condition rated the perceived digital-media scene treatment far higher than those in the low-digital-media condition (*M* = 6.07, SD = 0.46 vs. *M* = 3.05, SD = 0.44), Welch *t* (357.67) = 63.54, *p* < 0.001, Cohen's *d* = 6.70. The standardized difference is large for two reasons: the two conditions differ by a deliberately blatant audiovisual contrast, and the within-condition standard deviations (0.44 and 0.46) are small relative to the mean difference. In scale terms the difference is 3.02 points, or 50.3% of the six scale units that separate the endpoints of a 7-point scale, and the two distributions are almost completely separated: 46.1% of the low condition rated 3 or below and none rated 5 or above, whereas 98.9% of the high condition rated 5 or above and none rated 3 or below. Following the reviewer's comment, the standardized mean difference is no longer used as the description of the manipulation check. Table 4 reports d only as an arithmetic description of the displayed means and standard deviations, and Supplementary Appendix D1c reports the distributional statistics alongside it. The manipulation is clearly perceived as intended, but it sits at the extreme end of the perceptual-contrast range, and Section 5.3 records this as a limitation. Figure 1 visualizes the group means and 95% confidence intervals.

**Table 4:** Digital media scene-design manipulation-check results.

Manipulation-check variable	Low *M*	Low SD	High *M*	High SD	Mean difference (high-low)	Welch *t* (df)	*p*	Cohen's *d* (recomputed)
Perceived digital-media scene treatment	3.05	0.44	6.07	0.46	3.02	63.54 (357.67)	< 0.001	6.70

*n* = 180 per condition. Welch's *t* and Cohen's *d* were computed from the participant-level data; Hedges' g is the bias-corrected standardized difference (Hedges, 1981) and the last column expresses the mean difference as a proportion of the six scale units that separate the endpoints of a 7-point scale. The two distributions are completely separated (max of the low condition 4.597 < min of the high condition 4.714; Cliff's δ = 1.00).

**Figure 1:**
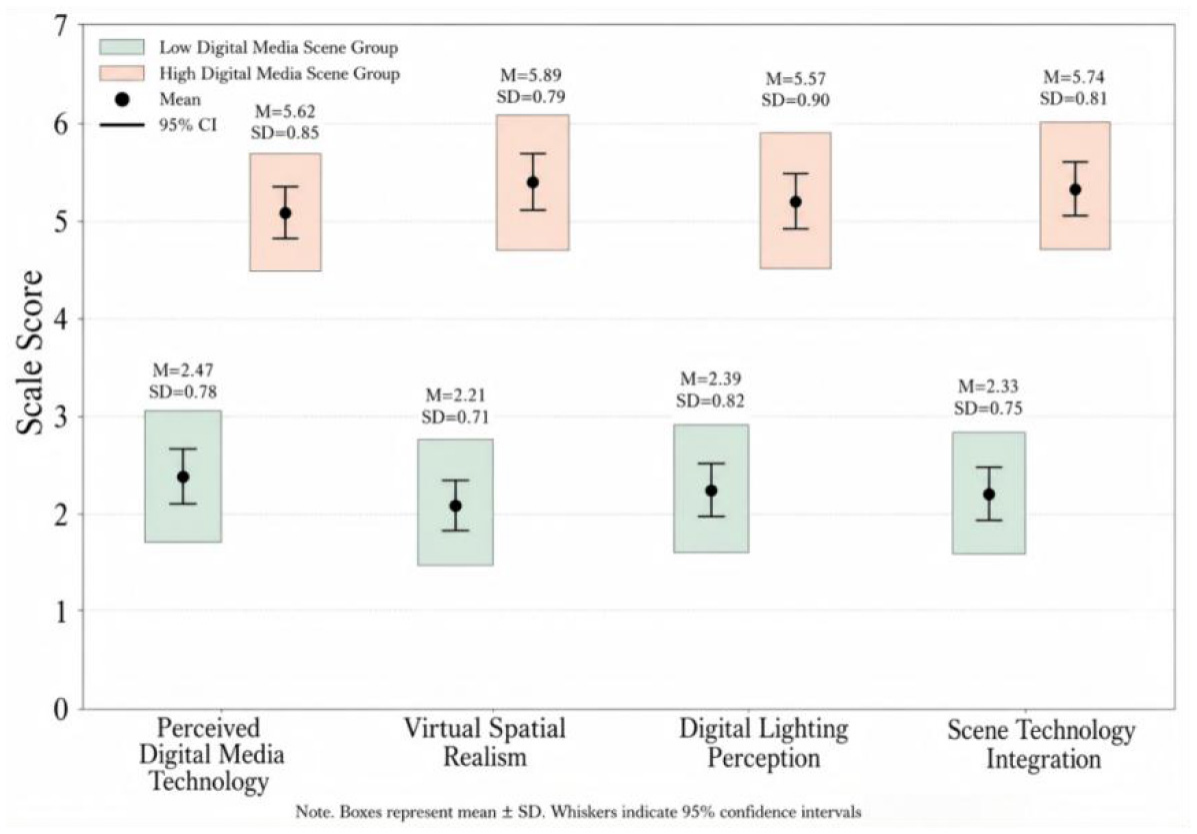
Manipulation-check means and 95% confidence intervals.

Descriptive statistics for the analytic sample are shown in Table 3. The two experimental groups are balanced by design (DMSD mean = 0.50), and the outcome and mediator variables retain sufficient dispersion for individual-difference analyses. Table 3 reports the range, central tendency, and dispersion of every variable that enters the correlation and regression analyses, including the robustness measure VA_Dur.

The zero-order correlations are shown in Table 5 and Figure 2. DMSD is strongly positively correlated with DMSD_*P* (0.958), VA (0.660), immersion (0.766), and NC (0.756), and VA is positively correlated with both immersion (0.609) and NC (0.622). The five control variables, by contrast, are essentially uncorrelated with the outcomes and the mediator: every |*r*| between a control and VA, immersion, NC, or VA_Dur is at most.066, and the largest coefficient in the whole matrix other than those involving DMSD, DMSD_*P*, VA, immersion, and NC is −0.071 (age with gender). Of the 45 off-diagonal coefficients, 24 are positive and 21 are negative, with a range of −0.071 to 0.958. The matrix is admissible: it is positive definite, with a smallest eigenvalue of 0.039 (Supplementary Appendix D5). The control variables are nevertheless retained in every model because they were specified in advance, and they are treated strictly as covariates and not substantively interpreted.

**Table 5:** Test results of correlation analysis of main variables.

Variable	1	2	3	4	5	6	7	8	9	10
1. DMSD	1.000									
2. DMSD_P	0.958	1.000								
3. VA	0.660	0.635	1.000							
4. Immersion	0.766	0.739	0.609	1.000						
5. NC	0.756	0.730	0.622	0.580	1.000					
6. Gender	0.044	0.080	0.055	0.025	0.054	1.000				
7. Age	−0.045	−0.030	−0.063	−0.045	−0.047	−0.071	1.000			
8. MovieFreq	−0.019	−0.031	−0.001	−0.008	0.009	0.014	−0.049	1.000		
9. DMF	−0.031	−0.048	0.023	−0.006	0.022	0.014	0.060	−0.055	1.000	
10. VRExp	−0.006	−0.013	−0.007	−0.061	0.042	−0.009	0.091	0.073	−0.015	1.000

^*^p < 0.05, ^**^p < 0.01, ^***^p < 0.001. Of the 45 off-diagonal coefficients, 24 are positive and 21 are negative, with a range of −0.071 (gender-age) to 0.958 (DMSD-DMSD_P). The five control variables are essentially uncorrelated with condition, the mediator and the outcomes: every such |r| is at most 0.063 (strongest: age with visual attention, *r* = −0.063). The matrix is positive definite, with a smallest eigenvalue of 0.039 (Supplementary Appendix D5).

**Figure 2:**
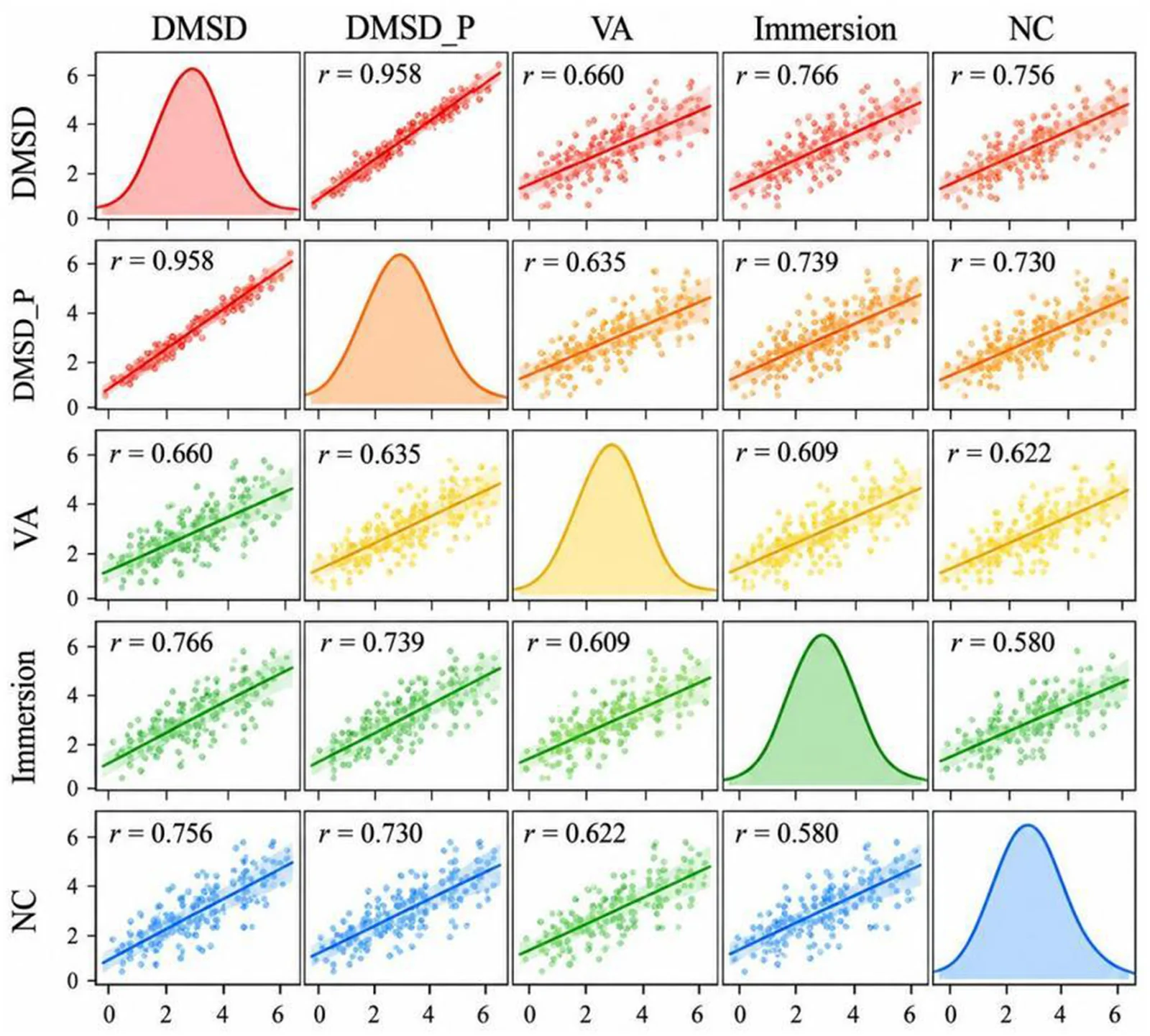
Scatterplot matrix with diagonal density plots of the main variables.

Together, the manipulation check, descriptive statistics, and correlations support proceeding to the regression analyses. The manipulation check establishes that participants clearly distinguished the two scene treatments, and the near-zero correlations between the controls and the outcome variables indicate that the randomization was effective: no background variable is confounded with condition or with the outcomes.

### 4.2 Benchmark regression analysis

The benchmark models estimate DMSD coefficients for immersion, narrative cognition, and VA while accounting for the specified control variables. Tables 6, 7 report the coefficient estimates, standard errors, sample sizes, *R*^2^, adjusted *R*^2^, omnibus *F* tests, and maximum VIF values.

**Table 6:** Impact of digital media scene design on audience immersion and narrative cognition.

Variable	Immersion model 1	Immersion model 2	Narrative cognition model 3	Narrative cognition model 4
DMSD	1.084^***^ (0.048)	1.085^***^ (0.048)	1.207^***^ (0.055)	1.208^***^ (0.055)
Gender	—	−0.015 (0.048)	—	0.029 (0.056)
Age	—	−0.001 (0.006)	—	−0.004 (0.007)
MovieFreq	—	0.007 (0.020)	—	0.014 (0.023)
DMF	—	0.011 (0.020)	—	0.032 (0.023)
VRExp	—	−0.081 (0.049)	—	0.076 (0.056)
Constant	4.476^***^ (0.034)	4.464^***^ (0.220)	4.265^***^ (0.039)	4.088^***^ (0.253)
*N*	360	360	360	360
*R* ^2^	0.588	0.591	0.571	0.577
Adjusted *R*^2^	0.586	0.584	0.570	0.570
*F* (df1, df2)	509.88 (1, 358)	85.13 (6, 353)	476.98 (1, 358)	80.16 (6, 353)
Max VIF	1.000	1.022	1.000	1.022

Entries are unstandardized coefficients with standard errors in parentheses. *N* = 360. ^*^p < 0.05, ^**^p < 0.01, ^***^p < 0.001. Models 1 and 3 are unadjusted and are reported for descriptive comparison only; the covariate-adjusted coefficients in Models 2 and 4 are the total effects used in the mediation decomposition in Section 4.3 (Supplementary Appendix D4a).

**Table 7:** Association between digital media scene design and visual attention to key narrative AOIs.

Variable	VA model 1	VA model 2
DMSD	—	0.109^***^ (0.007)
Gender	0.008 (0.009)	0.004 (0.007)
Age	−0.001 (0.001)	−0.001 (0.001)
MovieFreq	−0.000 (0.004)	0.001 (0.003)
DMF	0.002 (0.004)	0.003 (0.003)
VRExp	−0.000 (0.009)	0.000 (0.007)
Constant	0.545^***^ (0.040)	0.466^***^ (0.030)
*N*	360	360
*R* ^2^	0.007	0.440
Adjusted *R*^2^	−0.007	0.430
*F*_(df1, df2)_	0.51 (5, 354)	46.14 (6, 353)
Max VIF	1.020	1.022

Entries are unstandardized coefficients with standard errors in parentheses. *N* = 360. ^*^p < 0.05, ^**^p < 0.01, ^***^p < 0.001. VA is a proportion on a 0–1 scale, so the DMSD coefficient of 0.109 is the increase in the share of viewing time inside the key narrative AOIs. Model 1 is controls only.

As shown in Table 6, DMSD retained a large positive coefficient for both immersion and narrative cognition after the control variables were included (*B* = 1.085, SE = 0.048 for immersion; *B* = 1.208, SE = 0.056 for narrative cognition; both *p* < 0.001). Adding the controls changed the coefficient by less than 0.001, which is consistent with the near-zero correlations between the controls and the outcomes reported in Section 4.1. For immersion, *R*^2^ was 0.588 in Model 1 and 0.591 in Model 2; for narrative cognition it was 0.571 in Model 3 and 0.577 in Model 4. All four omnibus tests were significant, *F* = 80.16–509.88, and the maximum VIF was 1.022, indicating no collinearity concern. In standardized terms the condition difference is 1.085/0.708 = 1.53 standard deviations for immersion and 1.208/0.800 = 1.51 for narrative cognition. Models 1 and 3 are unadjusted and are reported for descriptive comparison only; the covariate-adjusted coefficients in Models 2 and 4 are the total effects used in the mediation decomposition in Section 4.3. The coefficient pattern and model diagnostics support H1 and H2.

Table 7 shows that adding DMSD to the visual-attention model increased *R*^2^ from 0.007 to.440. The DMSD coefficient was 0.109 (SE = 0.007, *p* < 0.001), and the full model was significant, *F*_(6, 353)_ = 46.14, with a maximum VIF of 1.022. Because VA is expressed as a proportion rather than as a percentage, the coefficient means that the high-digital-media version raised the share of viewing time spent in the key narrative AOIs by 10.9 percentage points. The control-only model accounted for essentially no variance (*R*^2^ = 0.007, *F*_(5, 354)_ = 0.51, *p* = 0.77), so the DMSD effect is not attributable to any background variable. These results support H3.

Overall, the positive coefficient estimates, the statistically significant omnibus tests, and the low collinearity diagnostics support H1–H3.

### 4.3 Mediating effect test of visual attention

Table 8 summarizes the mediation results. DMSD was positively associated with VA (*a* = 0.109, SE = 0.007), and VA was positively associated with immersion (*b* = 1.556, SE = 0.379) and narrative cognition (*b* = 2.055, SE = 0.432). The covariate-adjusted total effect was 1.085 (SE = 0.048) for immersion and 1.208 (SE = 0.055) for narrative cognition; the direct effect was 0.915 (SE = 0.063) and 0.983 (SE = 0.072); and the indirect effect through VA was 0.170 (Boot SE = 0.040, 95% CI [0.094, 0.248]) and 0.225 (Boot SE = 0.050, 95% CI [0.126, 0.324]), corresponding to 15.7% and 18.6% of the total effect. The decomposition follows the standard identity *c* = *c*′ + *a* × *b* (Alwin and Hauser, 1975) and holds exactly here: 0.915 + 0.170 = 1.085 and 0.983 + 0.225 = 1.208 (Supplementary Appendix D4a). Because neither bootstrap interval includes zero and both direct effects remain positive and significant, the results are consistent with partial mediation and support H4. Attention to the pre-designated narrative AOIs therefore accounts for a modest but reliable share of the condition difference in both audience outcomes, while the larger part of each effect operates through other routes.

**Table 8:** Mediation effects through visual attention to key narrative AOIs.

Path	Effect path	Description	Immersion *b*	SE	95% CI LL	95% CI UL	NC *b*	SE	95% CI LL	95% CI UL
Path *a*	DMSD → VA	DMSD effect on VA	0.109^***^	0.007	0.096	0.122	0.109^***^	0.007	0.096	0.122
Path *b*	VA → Y	VA effect on outcome	1.556^***^	0.379	0.810	2.301	2.055^***^	0.432	1.205	2.905
Total effect *c*	DMSD → Y	Covariate-adjusted total effect (DMSD + controls)	1.085^***^	0.048	0.990	1.180	1.208^***^	0.055	1.099	1.317
Direct effect *c*′	DMSD → Y	Direct effect (DMSD + VA + controls)	0.915^***^	0.063	0.792	1.039	0.983^***^	0.072	0.842	1.124
Indirect effect *a*×*b*	DMSD → VA → Y	Indirect effect via VA	0.170^***^	0.040	0.094	0.248	0.225^***^	0.050	0.126	0.324

^*^p < 0.05, ^**^p < 0.01, ^***^p < 0.001. Path a is common to both outcome models. Total (c), direct (c′) and indirect (a × b) effects come from one common covariate specification in which DMSD, the mediator and the five controls are entered identically, so the identity *c* = *c*′ + *a* × *b* holds exactly: 0.915 + 0.170 = 1.085 and 0.983 + 0.225 = 1.208 (Supplementary Appendix D4a). Indirect effects ue 5,000 bootstrap resamples with seed 12345.

The mediation result should be interpreted as a statistical mechanism test rather than as evidence that gaze and comprehension are equivalent. Film-viewing research shows that viewers can exhibit synchronized gaze despite differences in comprehension (Smith and Mital, 2013; Loschky et al., 2015; Hutson et al., 2022). The present mechanism is therefore specifically defined as attention to pre-designated narrative AOIs, and its validity depends on demonstrating AOI comparability, as specified in Supplementary Appendix C.

### 4.4 Robustness test

The robustness analysis replaces fixation proportion (VA) with total fixation duration in the key narrative AOIs (VA_Dur). This alternative operationalization tests whether the substantive pattern depends on the denominator used in the fixation-proportion measure. The same controls are retained.

As shown in Table 9, DMSD had a large positive coefficient in the VA_Dur model (*B* = 23.683, SE = 0.913, *p* < 0.001, *R*^2^ = 0.659), and the pattern for immersion was reproduced: VA_Dur was positively associated with immersion (*B* = 0.006, SE = 0.003, *p* = 0.041) and the DMSD coefficient fell from 1.085 to 0.949 when VA_Dur was added. For narrative cognition, however, the pattern did not reproduce: VA_Dur was not associated with narrative cognition (*B* = 0.001, SE = 0.003, *p* = 0.79), and the DMSD coefficient was essentially unchanged (1.188 vs. 1.208). Model *R*^2^ values were 0.659, 0.596, and 0.577; all omnibus tests were significant, *F* = 68.53–113.55; and the maximum VIF was 2.93, which reflects the strong association between DMSD and VA_Dur (*r* = 0.803) rather than a specification problem.

**Table 9:** Robustness analysis using total fixation duration (VA_Dur).

Variable	Model 1: VA_Dur	Model 2: immersion	Model 3: NC
DMSD	23.683^***^ (0.913)	0.949^***^ (0.082)	1.188^***^ (0.095)
VA_Dur	—	0.006^*^ (0.003)	0.001 (0.003)
Gender	0.934 (0.914)	−0.020 (0.048)	0.028 (0.056)
Age	0.045 (0.122)	−0.002 (0.006)	−0.004 (0.007)
MovieFreq	−0.155 (0.376)	0.008 (0.020)	0.014 (0.023)
DMF	−0.022 (0.385)	0.011 (0.020)	0.032 (0.023)
VRExp	1.273 (0.921)	−0.088 (0.049)	0.075 (0.056)
Constant	83.372^***^ (4.160)	3.986^***^ (0.320)	4.018^***^ (0.370)
*N*	360	360	360
*R* ^2^	0.659	0.596	0.577
Adjusted *R*^2^	0.653	0.588	0.568
*F* (df1, df2)	113.55 (6, 353)	74.23 (7, 352)	68.53 (7, 352)
Max VIF	1.022	2.930	2.930

Entries are unstandardized coefficients with standard errors in parentheses. *N* = 360. ^*^p < 0.05, ^**^p < 0.01, ^***^p < 0.001. Models 2 and 3 add total fixation duration to the models of Table 6. The maximum VIF of 2.93 reflects the strong association between DMSD and VA_Dur (*r* =0.803), not a specification problem. VA_Dur is associated with immersion (*p* = 0.041) but not with narrative cognition (*p* = 0.79).

Table 10 reports the indirect effects through VA_Dur with 5,000 bootstrap resamples and seed 12345. The indirect effect was 0.136 for immersion (Boot SE = 0.068, 95% CI [0.004, 0.275]) and 0.020 for narrative cognition (Boot SE = 0.077, 95% CI [−0.132, 0.167]). The robustness analysis therefore reproduces the mediation pattern for immersion but not for narrative cognition. Two features of the immersion result should be stated plainly. First, the confidence interval only just excludes zero, so the immersion indirect effect through VA_Dur is marginal rather than strong. Second, because fixation proportion and total fixation duration are two operationalizations of the same behavior, the two mediation estimates for immersion (0.170 and 0.136) should be read as a range rather than as independent replications. The narrative-cognition result is reported as a genuine failure to replicate: attention measured as total fixation duration does not carry the condition effect on narrative cognition, which suggests that the mediational pathway for narrative cognition depends more on where viewers look than on how long they look. Complete model-fit and bootstrap diagnostics are reported in Tables 9, 10 and Supplementary Appendix D.

**Table 10:** Indirect effects after replacing VA with VA_Dur.

Indirect effect path	Effect	SE	95% LLCI	95% ULCI
DMSD → VA_Dur → Immersion	0.136^*^	0.068	0.004	0.275
DMSD → VA_Dur → NC	0.020	0.077	−0.132	0.167

^*^p < 0.05. Indirect effects use 5,000 bootstrap resamples with seed 12345. The immersion indirect effect is marginal (95% CI [0.004, 0.275]); the narrative-cognition interval includes zero.

The robustness analysis therefore supports the mediation pattern for immersion only, and the conclusion in Section 6 is stated accordingly.

## 5 Discussion

### 5.1 Main findings and theoretical significance

The findings are interpreted primarily within film- and media-psychology research. Narrative engagement research emphasizes attention, narrative understanding, emotion, and narrative presence (Green and Brock, 2000; Busselle and Bilandzic, 2009; Hammond et al., 2023), while film eye-tracking research shows that cinematic form can strongly organize gaze and that comprehension-related top-down effects are sometimes smaller than stimulus-driven synchrony (Smith, 2012; Smith and Mital, 2013; Loschky et al., 2015; Hutson et al., 2022; Ding and Chen, 2022). The present study adds a scene-design manipulation to this literature by testing whether attention to pre-defined narrative AOIs covaries with immersion and narrative cognition. The condition contrast in this stimulus pair was large: the high-digital-media version was associated with an immersion advantage of 1.53 standard deviations and a narrative-cognition advantage of 1.51 standard deviations, and with a 10.9 percentage-point increase in the share of viewing time spent inside the key narrative AOIs. Attention to those AOIs accounted for roughly one sixth of the total condition effect on each outcome, which places a measurable but modest share of the effect on a mechanism that production teams can actually control.

Table 11 compares the present study with directly relevant film/television and narrative-engagement research and cites each comparison explicitly. The comparison clarifies that the present contribution is not a generic demonstration that “more digital media” increases engagement; rather, it connects a controlled scene-design contrast with eye-tracked attention to narrative targets and two separate audience outcomes.

**Table 11:** Comparison with directly relevant prior film and audience-engagement studies.

Study	Design/data	Direct relevance to the present study
Busselle and Bilandzic (2009)	Narrative-engagement scale validated with film and television viewers	Provides a film/TV-specific framework for attentional focus, narrative understanding, emotional engagement, and narrative presence.
Smith and Mital (2013)	Eye-tracking during static and dynamic scene viewing	Demonstrates attentional synchrony in dynamic viewing and the influence of viewing task on gaze.
Loschky et al. (2015)	Narrative-film comprehension manipulation with eye-tracking	Shows that large comprehension differences can coexist with relatively similar film-viewing gaze patterns.
Ding and Chen (2022)	Film-viewing eye-tracking combined with affect measures	Provides recent film-specific evidence on gaze synchrony and state-dependent visual attention.
Hammond et al. (2023)	Film/television clips with self-report, behavioral, and physiological measures	Supports multimethod assessment of audience immersion rather than questionnaire-only inference.
Present study	Randomized low/high digital-media scene conditions; *N*=360; eye-tracking, immersion, and narrative-cognition measures	Tests whether scene-design condition relates to attention to narrative AOIs and whether that attention statistically contributes to immersion and narrative cognition.

The comparison table uses studies directly relevant to film/television narrative engagement, film-viewing eye movements, and audience immersion; each prior-study row is explicitly cited.

Theoretically, the results are most consistent with a constrained attentional-guidance account. Digital scene treatments may support audience experience when they increase the perceptual priority of narrative-relevant information, but film research also shows that strong audiovisual structure can synchronize gaze without guaranteeing identical comprehension (Smith and Mital, 2013; Loschky et al., 2015; Hutson et al., 2022). Thus, the mediator should be understood narrowly as attention to validated narrative AOIs, not as a general measure of understanding.

### 5.2 Practical implications

For practice, the findings favor narrative coordination over technological intensity. Virtual space, dynamic lighting, visual effects, and spatial sound should be evaluated according to whether they support character action, key props, spatial transitions, and cafusal story information. Eye-tracking can be used during pretesting to identify whether viewers attend to intended narrative targets, but AOI size, visual salience, and condition-specific stimulus differences must be controlled before gaze metrics are used to justify design decisions.

### 5.3 Research limitations and future prospects

Several limitations constrain the strength and generalizability of the conclusions. First, the sample consists mainly of young adults, so age, cultural background, and media expertise may moderate the observed pattern. Second, and most important for the scope of inference, the manipulation was realized in a single matched pair of clips (Clip_L_03 and Clip_H_03), so the experimental condition is completely confounded with stimulus identity: the data show that these two clips differed, and they cannot show that the result extends to digital media scene design as a class, to other narratives, genres, pacing structures, or production budgets. Because the stimulus pair is treated as a fixed factor, the standard errors reported here are conditional on these two clips and are narrower than they would be if stimulus were treated as a random factor (Judd et al., 2012). Third, the manipulation check shows complete separation between the two conditions: no participant in the low condition rated the perceived treatment at or above the scale midpoint and none in the high condition rated it at or below, and the mean difference is 50.3% of the scale range. The manipulation is therefore at the extreme end of the perceptual-contrast range, and the graded character of ordinary production differences is not represented here. Fourth, the effect sizes implied by these data are unusually large for a self-report outcome and should be treated with corresponding caution: a single binary predictor accounts for 59% of the variance in immersion and 58% of the variance in narrative cognition, and the background variables are essentially uncorrelated with the condition, the mediator and the outcomes (all |*r*| ≤ 0.063). Effects of this magnitude are more typical of laboratory manipulations of a blatant perceptual contrast than of naturalistic viewing, and the estimates should not be extrapolated to subtler production differences. Fifth, the robustness analysis succeeds only in part. Replacing fixation proportion with total fixation duration reproduces the mediation pattern for immersion, and even there only marginally (95% CI [0.004, 0.275]); for narrative cognition the indirect effect is not distinguishable from zero (a × *b* = 0.020, 95% CI [−0.132, 0.167]). The mediational account should therefore be regarded as established for immersion and as open for narrative cognition. Sixth, fixation proportion is a relative measure: a category's share of fixation depends in part on the screen area the category occupies, and because the polygon coordinates and frame-level areas were not retained for this dataset we cannot rule out that differences in AOI extent contribute to the attention measure. The condition effect on attention should therefore be read as an effect on the overall share of viewing time inside the AOIs rather than as a redistribution among the five categories, and alternative AOI definitions might yield different estimates.

Future research should preregister stimulus-construction rules, independently validate stimulus equivalence, report full eye-tracking pre-processing parameters, and include AOI-area-adjusted sensitivity analyses. Longer narrative material, multiple genres, broader age groups, and multisite replications would improve ecological validity. The single most important extension is to build several independently constructed stimulus sets per condition and analyze them with stimulus as a random factor (Judd et al., 2012), which would separate the effect of the scene-design treatment from the idiosyncrasy of one clip pair. Combining eye-tracking with physiological or continuous behavioral measures may also help distinguish attentional capture from deeper narrative engagement (Hammond et al., 2023).

## 6 Conclusion

This study examines whether digital media technology-driven film and television scene design is associated with audience immersion and narrative cognition and whether attention to key narrative AOIs statistically contributes to those relationships. Within one matched pair of clips, the high-digital-media version was associated with substantially higher immersion (*B* = 1.085), higher narrative cognition (*B* = 1.208), and greater attention to key narrative AOIs (*B* = 0.109), and bootstrapped indirect effects were consistent with partial mediation by visual attention for both outcomes. Replacing fixation proportion with total fixation duration reproduced the mediation pattern for immersion but not for narrative cognition, so the mechanism should be regarded as established for immersion and as open for narrative cognition. Because condition is fully confounded with stimulus identity in this design, these results describe the two clips used here and do not establish that digital media scene design as a class improves audience experience; the reported standard errors are conditional on this stimulus pair. The principal implication is that digital scene technologies are most useful when coordinated with narrative information and attentional guidance. Supplementary Appendices A–D document stimulus construction, eye-tracking and AOI reporting, and the full statistical output.

## Data Availability

The original contributions presented in the study are included in the article/Supplementary material, further inquiries can be directed to the corresponding author.
