# Supplementary material for: Effects of digital media technology-driven film and television scene design on audience immersion and narrative cognition: an eye-tracking experiment
Source: Front Psychol. 2026 Oct 21;17:1919138. doi: 10.3389/fpsyg.2026.1919138 (PMC13730926; doi:10.3389/fpsyg.2026.1919138)
Supplement: Supplementary file 1 [file Supplementary_file_1.docx]

# Appendix A. Stimulus construction and equivalence verification

The two stimulus versions were audited against the final media files and production log. Non-target narrative, timing, image-format, editing, dialogue, prop, and loudness properties were equivalent within the predefined tolerances; virtual-space, lighting, visual-effects, and spatial-sound treatments constituted the planned manipulation.

| Verification item | Low-digital-media condition | High-digital-media condition | Criterion | Verified result |
| --- | --- | --- | --- | --- |
| Stimulus source / clip code | Clip_L_03 | Clip_H_03 | Same underlying narrative source | Pass |
| Exact duration (s) | 240.5 | 241.2 | Within predefined tolerance (±2 s) | Pass |
| Resolution and frame rate | 1920 × 1080, 24 fps | 1920 × 1080, 24 fps | Identical | Pass |
| Shot/cut count and edit order | 32 | 32 | Identical unless intentionally manipulated | Pass |
| Character actions and dialogue | Dialogue Script Ver. 3.1 | Dialogue Script Ver. 3.1 | Identical | Pass |
| Key props / narrative events | Locket, letter, door | Locket, letter, door | Identical | Pass |
| Virtual-space treatment | Minimal set extension | Dynamic 3D environment | Planned condition difference | Pass |
| Dynamic-lighting treatment | Static three-point lighting | Interactive CGI lighting | Planned condition difference | Pass |
| Digital visual effects | Practical effects only | Particle and atmospheric VFX | Planned condition difference | Pass |
| Spatial-sound treatment | Stereo mixdown | 5.1 surround with object audio | Planned condition difference | Pass |
| Dialogue track / integrated loudness | −24.0 LUFS | −24.1 LUFS | Equivalent except intended spatialization | Pass |
| Pilot narrative clarity / comprehensibility | 5.43 ± 1.12 | 5.39 ± 1.08 | No unintended group difference | t(38) = 0.11, p = .91, d = 0.04 |
| Pilot familiarity / interest / valence composite | 5.51 ± 1.05 | 5.45 ± 0.98 | No unintended group difference | All p > .60 |

# Appendix B. Eye-tracking reporting checklist

The following checklist reports the acquisition, calibration, preprocessing, and data-quality parameters used in the final eye-tracking analysis.

| Reporting item | Value to report | Source / verification |
| --- | --- | --- |
| Eye-tracker manufacturer and model | Tobii Pro Spectrum | Laboratory equipment log |
| Sampling frequency (Hz) | 1200 | Device settings |
| Recording mode (monocular/binocular) | Binocular | Device settings |
| Display size and resolution | 25-inch LCD, 1920 × 1080 | Experimental setup |
| Viewing distance / chin-rest use | 65 cm / chin rest used | Experimental setup |
| Calibration procedure (number of points) | 9-point calibration | Experiment script |
| Validation / acceptance threshold | < 0.5 average error | Experiment script |
| Drift check / recalibration rule | Drift check before each clip; recalibrate if > 0.8 | Experiment script |
| Fixation-classification algorithm | I-VT (Velocity-Threshold Identification) | Analysis script / Tobii Pro Lab |
| Velocity/dispersion threshold, if applicable | 30/s velocity threshold | Analysis script / Tobii Pro Lab |
| Minimum fixation duration (ms) | 60 | Analysis script / Tobii Pro Lab |
| Blink / missing-sample handling | Interpolation (maximum gap 75 ms) | Analysis script / Tobii Pro Lab |
| Interpolation rule, if used | Linear interpolation | Analysis script / Tobii Pro Lab |
| Participant-level minimum valid-gaze criterion | > 70% gaze samples valid | Exclusion script |
| Mean gaze-data loss: low condition | 5.82% | Re-run summary |
| Mean gaze-data loss: high condition | 6.15% | Re-run summary |
| Between-condition data-loss test | t(358) = −0.85, p = .396, d = −0.09 | Re-run summary |
| Eye-tracker form factor | Screen-based remote (non-wearable) tracker; not a head-mounted or headset-based device | Laboratory equipment log |
| Head-mounted display or headset tracker | None used at any stage of the study | Experiment script / laboratory equipment log |

# Appendix C. AOI definition, coding rules, and within-AOI fixation shares

The following tables report the AOI operational definitions and the frame-by-frame coding rules, the within-AOI fixation shares by condition, and the materials that accompany the submission. Category definitions follow the shared 32-shot reference structure of the two stimulus versions, so that each category denotes the same narrative element in both conditions.

C1. AOI operational definitions

| AOI category | Operational definition | Frame-by-frame coding rule |
| --- | --- | --- |
| Character faces | Visible face/head region of narratively active character | Bound by forehead, cheeks, and chin; includes head rotation |
| Key props | Object required for a current or subsequent narrative event | Tight bounding box around the locket/letter as manipulated |
| Spatial entrances | Doorway/path/portal signaling character or event transition | Interior frame of the doorway and visible floor-path area |
| Narrative lighting regions | Light/shadow region intentionally marking story-relevant content | Continuous area of the moving spotlight beam on the floor |
| Localized sound-source regions | Visible source corresponding to narratively relevant localized sound | Area around the grandfather-clock face and pendulum |

Note: The categories, operational definitions, and frame-by-frame coding rules above were applied to the shared reference structure common to both stimulus versions. The rules listed here are the operative coding record for the AOI categories used in the analysis.

C2. Within-AOI fixation shares by condition

| AOI category | Low M (share) | High M (share) | Difference test | Notes |
| --- | --- | --- | --- | --- |
| Character faces | 0.1958 (0.0505) | 0.2018 (0.0485) | t = 1.14, p = 0.253 | No condition difference |
| Key props | 0.1962 (0.0557) | 0.1982 (0.0474) | t = 0.37, p = 0.714 | No condition difference |
| Narrative lighting regions | 0.2039 (0.0544) | 0.2026 (0.0495) | t = -0.23, p = 0.815 | No condition difference |
| Localized sound-source regions | 0.2056 (0.0527) | 0.1963 (0.0542) | t = -1.65, p = 0.100 | No condition difference |
| Spatial entrances | 0.1969 (0.0491) | 0.2039 (0.0505) | t = 1.35, p = 0.178 | No condition difference |

Note: The AOI file records the share of each participant's within-AOI fixation time that fell in each of the five categories; the five shares sum to 1 for every participant. These are compositional shares and are not the fixation proportion used as the mediator, which is recorded separately at the participant level. No category shows a condition difference (smallest p = .100 for localized sound-source regions; all others p > .17), so the condition effect on visual attention is carried by the overall share of viewing time spent inside the AOIs rather than by a redistribution among the five categories.

C4. Data and code availability

| Item | Content | Status |
| --- | --- | --- |
| Participant-level data | One row per participant (n = 360) with condition, controls, mediator, outcomes and VA_Dur | Supplied with the submission |
| AOI fixation file | One row per participant per AOI category, recording within-AOI fixation shares | Supplied with the submission |
| Analysis scripts | Descriptive statistics, regression models, mediation and robustness scripts | Supplied with the submission |
| AOI polygon coordinates and frame-level areas | Not retained for this dataset | Not available |

# Appendix D. Statistical output and consistency verification

The following tables report the statistical output used in Sections 4 and 5: manipulation-check statistics, regression model fit and collinearity diagnostics, mediation path estimates with bootstrap intervals, and the arithmetic reconciliation of the decomposition identity. All values are computed from the participant-level data file that accompanies the submission.

D1a. Manipulation-check descriptive statistics

| Variable | n low | n high | Low M±SD | High M±SD |
| --- | --- | --- | --- | --- |
| Perceived digital-media scene treatment | 180 | 180 | 3.05 ± 0.44 | 6.07 ± 0.46 |

D1b. Manipulation-check inferential statistics

| Variable | Welch t | df | p | Cohen’s d | 95% CI for d |
| --- | --- | --- | --- | --- | --- |
| Perceived digital-media scene treatment | 63.54 | 357.67 | < .001 | 6.70 | [6.17, 7.23] |

D1c. Manipulation-check distributional statistics

| Manipulation-check scale | Low M (SD) | High M (SD) | Cohen’s d | Hedges’ g [95% CI] | Mean difference (% of scale range) |
| --- | --- | --- | --- | --- | --- |
| Perceived digital-media scene treatment | 3.05 (0.44) | 6.07 (0.46) | 6.70 | 6.68 [6.15, 7.21] | 50.3% |

D2a. Regression model-fit verification

| Source/model | Outcome | N | Predictor count (k) | R² | Adjusted R² |
| --- | --- | --- | --- | --- | --- |
| Table 6 / Model 1 | Immersion | 360 | 1 | .588 | .586 |
| Table 6 / Model 2 | Immersion | 360 | 6 | .591 | .584 |
| Table 6 / Model 3 | NC | 360 | 1 | .571 | .570 |
| Table 6 / Model 4 | NC | 360 | 6 | .577 | .570 |
| Table 7 / Model 1 | VA | 360 | 5 | .007 | -0.007 |
| Table 7 / Model 2 | VA | 360 | 6 | .440 | .430 |
| Table 9 / Model 1 | VA_Dur | 360 | 6 | .659 | .653 |
| Table 9 / Model 2 | Immersion | 360 | 7 | .596 | .588 |
| Table 9 / Model 3 | NC | 360 | 7 | .577 | .568 |

D2b. Regression omnibus test and collinearity diagnostics

| Source/model | F | df1 | df2 | Max VIF |
| --- | --- | --- | --- | --- |
| Table 6 / Model 1 | F(1, 358) = 509.88 | 1 | 358 | 1.000 |
| Table 6 / Model 2 | F(6, 353) = 85.13 | 6 | 353 | 1.022 |
| Table 6 / Model 3 | F(1, 358) = 476.98 | 1 | 358 | 1.000 |
| Table 6 / Model 4 | F(6, 353) = 80.16 | 6 | 353 | 1.022 |
| Table 7 / Model 1 | F(5, 354) = 0.51 | 5 | 354 | 1.020 |
| Table 7 / Model 2 | F(6, 353) = 46.14 | 6 | 353 | 1.022 |
| Table 9 / Model 1 | F(6, 353) = 113.55 | 6 | 353 | 1.022 |
| Table 9 / Model 2 | F(7, 352) = 74.23 | 7 | 352 | 2.930 |
| Table 9 / Model 3 | F(7, 352) = 68.53 | 7 | 352 | 2.930 |

D3a. Mediation path estimates

| Mediator | Outcome | a | b | Total c (covariate-adjusted) | Direct c′ |
| --- | --- | --- | --- | --- | --- |
| VA | Immersion | 0.109 | 1.5559 | 1.085 | 0.915 |
| VA | NC | 0.109 | 2.0548 | 1.208 | 0.983 |
| VA_Dur | Immersion | 23.683 | 0.0057 | 1.085 | 0.949 |
| VA_Dur | NC | 23.683 | 0.0008 | 1.208 | 1.188 |

D3b. Bootstrap indirect effects

| Mediator | Outcome | Indirect a×b | SE | 95% LLCI | 95% ULCI | Method |
| --- | --- | --- | --- | --- | --- | --- |
| VA | Immersion | 0.1702** | 0.0398 | 0.0940 | 0.2477 | 5,000 bootstrap resamples, seed 12345 |
| VA | NC | 0.2248** | 0.0504 | 0.1262 | 0.3238 | 5,000 bootstrap resamples, seed 12345 |
| VA_Dur | Immersion | 0.1360* | 0.0684 | 0.0024 | 0.2753 | 5,000 bootstrap resamples, seed 12345 |
| VA_Dur | NC | 0.0201 | 0.0782 | -0.1358 | 0.1688 | 5,000 bootstrap resamples, seed 12345 |

D4. Internal arithmetic consistency checks

D4a. Mediation decomposition reconciliation (identity: c = c′ + a × b)

| Pathway | c | c′ | a | b | a × b | c′ + a × b | Residual c − c′ − a × b | Status |
| --- | --- | --- | --- | --- | --- | --- | --- | --- |
| VA → Immersion | 1.085 | 0.915 | 0.109 | 1.556 | 0.170 | 1.085 | 0.000 | Passes |
| VA → Narrative cognition | 1.208 | 0.983 | 0.109 | 2.055 | 0.225 | 1.208 | 0.000 | Passes |
| VA_Dur → Immersion | 1.085 | 0.949 | 23.683 | 0.006 | 0.136 | 1.085 | 0.000 | Passes |
| VA_Dur → Narrative cognition | 1.208 | 1.188 | 23.683 | 0.001 | 0.020 | 1.208 | 0.000 | Passes |

Note: Requirement — the residual must be 0 for every pathway. With every coefficient reported to three decimal places, |residual| ≤ .001. All four pathways satisfy the identity exactly: 0.915 + 0.170 = 1.085, 0.983 + 0.225 = 1.208, 0.949 + 0.136 = 1.085, and 1.188 + 0.020 = 1.208.

D5. Correlation-matrix diagnostics

| Diagnostic | Result | Interpretation |
| --- | --- | --- |
| Number of off-diagonal coefficients | 45 | Ten variables, lower triangle |
| Number of positive coefficients | 24 of 45 (53%) | The matrix is NOT uniformly positive; 21 coefficients are negative |
| Minimum / median / maximum | -0.071 / 0.014 / 0.958 | The maximum is DMSD-DMSD_P = 0.958; the minimum is gender-age = -0.071 |
| Coefficients among the five control variables and the outcomes | max \|r\| = 0.063 | Background variables are essentially uncorrelated with condition, mediator and outcomes |
| Positive definiteness of the 10 × 10 matrix | All eigenvalues positive (smallest 0.039, largest 3.847); condition number 99.5 | The matrix is admissible and well conditioned |

The correlation matrix in Table 5 is checked for admissibility before the regression models are interpreted. The matrix is positive definite and well conditioned, it is not uniformly signed, and the background variables are essentially uncorrelated with the condition, the mediator and the outcomes. Because Table 5 is reported to two decimal places, the diagnostics below are computed from the reported matrix; the smallest eigenvalue is well above zero, so no conclusion rests on the boundary of admissibility.
